# Cerebral Infarction in an Elderly Patient with Coronavirus Disease

**DOI:** 10.1590/0037-8682-0307-2020

**Published:** 2020-06-03

**Authors:** Handan Alay, Fatma Kesmez Can, Elif Gözgeç

**Affiliations:** 1 Ataturk University, Faculty of Medicine, Department of Infectious Diseases and Clinical Microbiology, Erzurum, Turkey.; 2 Ataturk University, Faculty of Medicine, Department of Radiology, Erzurum, Turkey.

**FIGURE 1:**
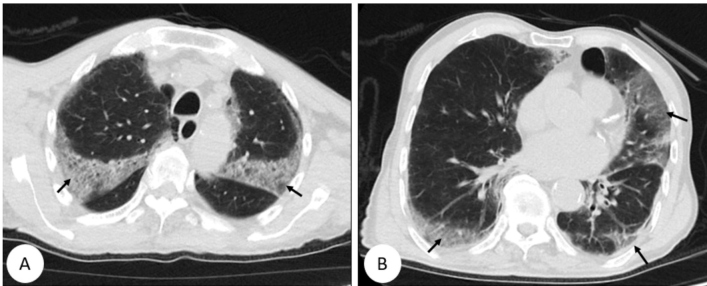
Axial section non-contrast computed tomography showing widespread ground-glass opacities and crazy paving patterns in the bilateral lungs (arrows).

**FIGURE 2:**
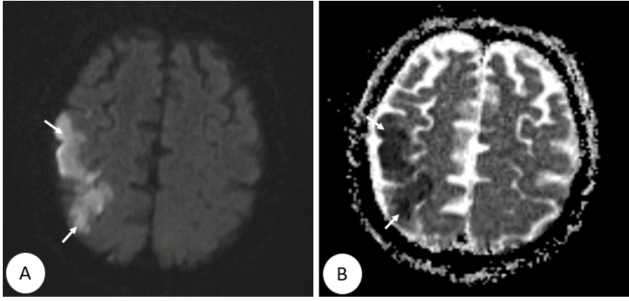
Brain diffusion magnetic resonance imaging showing areas of restricted diffusion compatible with hyperintense infarction in the right frontal lobe **(A)**, and hypointense infarction on apparent diffusion coefficient mapping **(B)** (arrows).

An 82-year-old man presented with cough and weakness and admitted to our clinic. His body temperature was 38^0^ C, heart rate 93 beats/min, respiratory rate 22 breaths/min, blood pressure 100/60 mmHg, and oxygen saturation 86% (oxygen mask 5 L/min). His blood leukocyte, neutrophil, lymphocyte, D-dimer, fibrinogen, c-reactive protein, ferritin, and procalcitonin levels were 8.56 x 10^3^/µL, 7.4x10^3^/µL, 0.62x 10^3^/µL, 2304 ng/mL, 638 mg/dL, 183 mg/L, 720 ng/mL, and 0.2 ng/mL, respectively. Computed tomography of the thorax revealed a suspected diagnosis of coronavirus disease (COVID-19) (Figure 1). Antiviral (Favipavir 2 x 1600 mg loading, 2 x 600 mg maintenance) and antibacterial (levofloxacin 500 mg/day) therapies were initiated. The patient’s oronasopharyngal swab specimen was positive for severe acute respiratory syndrome coronavirus 2 nucleic acid. Weakness and loss of muscle tone developed in the left arm on day 3 of treatment. Brain diffusion magnetic resonance imaging showed multiple advanced stage infarctions (Figure 2). Enoxaparin 0.5 mg/kg once every 12 hours and acetylsalicylic acid 100 mg 1x1 were added to treatment. The laboratory parameters improved. The patient was discharged on day 20.

COVID-19 can result in cerebral infarction and death in the elderly^1^ ^,^ ^2^. Anticoagulants are useful in elderly patients with high D-dimer due to the risk of coagulation dysfunction and cerebral infarction^3^. Thromboembolic complications must be considered in COVID-19 patients with known risk factors and abnormal laboratory findings.
